# Feasibility of a musculoskeletal ultrasound intervention to improve adherence in juvenile idiopathic arthritis: a proof-of concept trial

**DOI:** 10.1186/s12969-018-0292-3

**Published:** 2018-11-22

**Authors:** Leslie A. Favier, Tracy V. Ting, Avani C. Modi

**Affiliations:** 1 Cincinnati Children’s Hospital Medical Center, Department of Pediatric Rheumatology, 3333 Burnet Ave, MLC 4010, Cincinnati, OH 45229 USA; 2 Cincinnati Children’s Hospital Medical Center, Behavioral Medicine and Clinical Psychology, Center for Adherence and Self-Management, 3333 Burnet Ave, MLC 7039, Cincinnati, OH 45229 USA

**Keywords:** Adherence, Juvenile idiopathic arthritis, Musculoskeletal ultrasound

## Abstract

**Background:**

Non-adherence is a prevalent and modifiable issue in juvenile idiopathic arthritis (JIA) that currently lacks provider-based intervention. Education surrounding disease status is one way in which families remain engaged in their care. Musculoskeletal ultrasound is one such form of demonstrative, real-time education that may impact the way patients and caregivers self-manage their disease. The aims of this study are to 1) assess the feasibility, acceptability and perceived usefulness of musculoskeletal ultrasound as a non-adherence intervention tool and 2) to examine changes in methotrexate adherence in adolescents with JIA following the ultrasound.

**Methods:**

Eight adolescents with polyarticular or extended oligoarticular JIA and their caregivers completed this 12 week study. A within subject design was used to compare baseline and post-intervention adherence, quality of life and disease activity indices. Adherence measures included electronic measurement of methotrexate in addition to self-reported adherence questionnaires. The ultrasound intervention included a one-time, rheumatologist provided, educational examination of three or more currently or historically active joints.

**Results:**

The ultrasound intervention was found to be both feasible and acceptable. One hundred percent of eligible participants completed the ultrasound intervention. The ultrasound was well received by patients and caregivers, with most believing this to be a helpful tool. Baseline adherence was 75.3% among participants, with half of the participants being classified as non-adherent. Electronically measured and self-reported adherence measures did not show significant changes during the post-intervention period. Two participants improved, four participants maintained, and two participants decreased adherence. On ultrasound, 18/27 (66.7%) of the examined joints displayed abnormalities, with 63% being discrepant and additive to the rheumatologist’s physical examination.

**Conclusions:**

While our intervention did not show any changes in adherence, quality of life or disease activity indices in this proof-of-concept trial, the intervention does show promise in acceptability measures and merits future study in a more robust trial design. An additional study benefit was that the musculoskeletal ultrasound intervention was able to demonstrate subclinical disease, leading to clinically impactful therapeutic changes in several participants.

## Background

Juvenile Idiopathic Arthritis (JIA) is the most common rheumatic condition affecting children worldwide, with an estimated prevalence rate of 132 per 100,000 [1]. The disease spectrum includes classification into seven subtypes based on the number of joints affected and differences in symptom development and prognosis. Disease control is based on management of inflammation and progression of disease while limiting disability and deformity over time. The treatment regimen for JIA is multifaceted and includes oral and/or injectable medications, physical and occupational therapies, and some lifestyle modifications. Studies focused on patients with polyarticular JIA indicate that early and aggressive therapeutic approaches increased the likelihood of attainment and maintenance of disease remission [2, 3]. Nevertheless, even after remission is attained, the current risk of disease recurrence reaches approximately 40%, which in part may be due to waning treatment adherence [4].

Adherence is defined as the extent to which a person’s behavior follows healthcare treatment recommendations [5]. Factors that contribute to poor adherence include delayed benefits in early treatment, need for consistency over a long period of time, and negative side effects [6]. Unfortunately, poor adherence in JIA impacts health-related quality of life [7] and rates of active disease control [8]. For example, prescription refills for adherence to disease modifying anti-rheumatic drugs (DMARD) and injectable anti-tumor necrosis-alpha drugs only occurred 46.9 and 65.7% of the time, respectively [9]. Our prior work studying adherence barriers in the current treatment landscape indicates that 70% of adolescents with JIA and 77% of their caregivers endorse at least one adherence barrier [10]. The most commonly reported barriers include worry about future consequences, pain, and forgetting, which are all amenable to intervention. In fact, recent meta-analyses have demonstrated that healthcare provider-based adherence interventions have greater effects on adherence compared to other multi-component interventions [11]. To date, there are no evidence-based adherence promotion interventions within standard clinical rheumatology practice and this current study sought to establish proof-of-concept for a rheumatologist-provided intervention.

Musculoskeletal ultrasound (MSKUS) is an emerging form of disease surveillance that an increasing number of rheumatology providers are utilizing in clinical practice. Symptoms of chronic arthritis due to synovial hypertrophy and fluid accumulation within the joint space can accurately be identified in real-time during routine clinic visits [12, 13]. Ultrasound studies are efficient in both time and cost expenditure, as compared to the imaging gold standard MRI, and are able to spare the use of radiation or intravenous (IV) contrast burden [12]. Some evidence has suggested that subclinical disease activity including synovitis can be identified by the use of MSKUS surveillance [14–16]. MSKUS provides a longitudinal, objective characterization of disease status that allows an important opportunity for patient inclusion via demonstrative real-time education. Specifically, visual demonstration modalities reduce limitations imposed by healthcare literacy variation and possibly may better equip patients with knowledge regarding their disease [17]. Additionally, MSKUS may foster increased patient-provider interaction through shared disease visualization and discussion. Studies assessing adults with rheumatoid arthritis (RA) revealed that those who underwent ultrasound evaluation had more confidence in their ability to complete treatment recommendations [18, 19].

As the routine use of ultrasound increases in pediatric rheumatology, our aim in this proof-of-concept study is to assess the impact of MSKUS on adherence in adolescents with polyarticular JIA. This pilot study serves to efficiently establish the merit for more rigorous testing of the intervention. Our hypothesis was that MSKUS would be rated as feasible and acceptable to patients and parents and that following receipt of an MSKUS intervention, participants would demonstrate improvement in electronically-monitored and patient-reported adherence measures.

## Methods

### Study population

Participants were recruited from a rheumatology clinic at a Midwestern children’s hospital. Inclusion criteria were as follows: (1) adolescents, ages 10 to 17 years of age and their primary caregiver; (2) diagnosed with polyarticular or extended oligoarticular JIA and (3) taking methotrexate (oral or injectable) as part of their arthritis treatment regimen. Pertinent exclusion criteria included: (1) patients who were undergoing treatment for other chronic rheumatic conditions, (2) patients with other underlying chronic medical conditions involving daily prescribed therapy (i.e. Diabetes) and (3) patients with the diagnosis of significant developmental disorders impacting the ability to complete a questionnaires or understand a MSKUS intervention.

### Study design

This proof-of-concept study used a within-subject design where subjects act as their own control in a pre-post intervention comparison. The study time-line consisted of three total visits. At enrollment, participants were given an electronic adherence monitor to use for the entire study period. At baseline, demographic screening, patient/parent reported adherence questionnaires and disease-specific chart review were collected. Two to three-months later, participants received the intervention (i.e. MSKUS) and completed questionnaires to ensure no significant changes in outcomes of interest prior to the intervention. An additional two to three-months following the intervention, electronic adherence was recorded and repeated outcome measures were collected in addition to a feasibility and acceptability questionnaire. Study visits were scheduled along with regularly scheduled rheumatology clinic appointments, so there was some natural variation in the three-visit study timeline between patients. While three adolescent-parent dyads completed the final study questionnaires remotely and returned via mailer, the remainder of the sample completed all materials face-to-face. The relative small scale of this study serves to test this novel intervention using both a convenient sample and study visit timeline, with the intention of performing a more rigorous trial design in the future.

### Primary outcome measures

Primary outcome measures focused on the implementation of the intervention which included: 1) enrollment and retention rates, 2) proportion of completed questionnaires, 3) ability to utilize and return the electronic adherence monitoring device, and 4) acceptability of the ultrasound intervention as characterized by a written questionnaire. Caregivers and adolescents were asked to complete a study-specific questionnaire assessing feasibility and acceptability of the adherence intervention. Similar questionnaires have been used by adherence researchers and provide critical information on ways to improve upon the adherence intervention [20]. The measure included 9 items, demonstrated in Table 2. The questionnaire includes items related to the format, content, convenience, as well as perceived impact of the ultrasound intervention on outcomes. These items were scored on a 5-point Likert scale, with a score of 4 or 5 denoting a high degree of acceptability (i.e. agree or strongly agree).

### Secondary outcome measures

Secondary outcomes were designed to assess effectiveness of the intervention as an adherence promoter. The *Medication Event Monitoring Systems (MEMS)*, made by AARDEX Corporation, is an electronic monitoring system that measures the dosing histories of patient prescribed oral medications. It has two components: a standard plastic bottle with a threaded opening and a closure for the vial that contains a micro-electronic circuit to register the dates and times the bottle is opened and closed. Based on its reputation as the most prevalent adherence tracking device, with strong correlations to both pharmacy refill data and serum assay adherence measures, it was used to monitor adherence to methotrexate for the current study [21, 22]. The bottles used accommodated both pill and injectable vial form of methotrexate and this represents the first time that adherence to an injectable DMARD has been quantitatively measured in JIA. The MEMS TrackCap stores times and dates and the data can be transferred to a Windows-based computer. Data from the MEMS TrackCap was downloaded during the intervention and post-intervention study visit. Adolescents and caregivers were asked at study visits if there are any situations or times that the cap was not used to account for intentional dosing breaks or illnesses that may have prohibited dosing, and the data were edited to reflect these changes. Notably, our baseline assessment was 2–3 months in length, giving sufficient time for typical reactivity (e.g. 2–3 weeks) to using the monitor [23].

Adherence questionnaires included the *Parent Adherence Report Questionnaire (PARQ) and Child Adherence Report Questionnaire (CARQ)*. These JIA-specific tools measure beliefs and behaviors related to adherence to various prescribed JIA therapies. The tool is composed of parent and patient specific forms including 10 and 8 questions respectively, using a visual analog scale. Rasch analysis of the parent form demonstrated validity and reliability of the instrument [24, 25]. Currently, there are no scoring criteria for this tool and items are examined individually. For our study purpose, the item asking “how often do you follow your medication treatment” was used to ascertain self-reported adherence, for the parent and child separately.

Barriers to adherence were measured via a JIA-specific barriers assessment tool previously reported by our group, *Barriers to Adherence Tool (BAT)* [10]. This fourteen item checklist assesses logistical, social, psychological and knowledge-based adherence barriers in both patients and caregivers for all treatment modalities (medications, injections, infusions and physical/occupational therapy). While validation of this tool has not been formally undertaken in JIA, the themes represented by this measure have been systematically validated in other chronic pediatric conditions [26–28].

Baseline measures of current and historical disease activity, treatment regimens, and demographics were obtained at the time of study recruitment. Disease activity parameters extracted from the medical record included physical examination (including active joint counts), duration of disease, serologies and inflammatory indices (i.e. sedimentation rate and C-reactive protein) if available. Additionally, the patient and provider global assessment scores were used which include a visual analog log (VAS) score assessment of the patients overall well-being (Range 0–10, 10 being poor). These disease related measures were used to assess overall disease activity via the cJADAS (clinical juvenile disease activity score) scoring system [29]. The cJADAS is a validated for clinical and research use in JIA.

*Pediatric Quality of Life Inventory (PedsQL™)* Rheumatology Module is a disease-specific quality of life measure that assesses pain and hurt, daily activities, treatment, worry and communication. For the purpose of this study, the total score was used. Reliability for the total scale is 0.93 for the parent proxy report [30].

JIA-specific knowledge about disease and prescribed therapies was assessed by a tool designed for use in this study. This tool contains 11 items to measure baseline knowledge. All items were true/false for ease of completion for the adolescent. Themes addressed in the questionnaire included JIA comorbidities (i.e. eye disease), common therapies (i.e. immunosuppressants), aspects of JIA care (i.e. the need for physical therapy, lab testing and joint exams), and some items specific to methotrexate dosing (i.e. folic acid supplementation and nausea as an adverse effect). This tool is available upon request of corresponding author (LF).

### Ultrasound intervention

The ultrasound intervention was designed to be succinct and complementary to standard rheumatology care. An introduction to joint anatomy, physical disease manifestations and a brief synopsis of ultrasound imaging was provided to each family. The ultrasound included assessment of three or more historically active or symptomatic joints as demonstrated via prior provider physical examination. The ultrasound assessed at minimum 2 views (in orthogonal planes) of every joint evaluated; via both grayscale and Power Doppler (PD) modes. Standardized guidelines for image acquisition in JIA are limited [31]; joint specific images were obtained using recommendations for the knee joint by Ting et al. and other joints by Collado et al. [32] Determination of abnormal effusion, synovial hypertrophy and Power Doppler enhancement was completed in accordance to consensus guidance for synovitis in pediatrics [33]. PD mode was chosen over color Doppler based on optimal performance of the machines used at our center. Patient and caregiver were provided with verbal review of the findings and they were encouraged to participate in direct visualization of the images in real-time. No other specifics of their care (i.e. treatments) were verbally discussed at the time of the ultrasound, except in the case of one patient who was treated by the ultrasound interventionist. Each intervention took on average 20 min. Ultrasounds were performed by clinicians (LF, TT) with training levels of 2–6 years in MSKUS. No ultrasonographer blinding was pursued in this study. Abnormal findings based on the ultrasound were communicated to the patient’s primary rheumatologist.

### Statistical analysis

Adherence was calculated as the number of doses taken/number of doses prescribed *100%. Adherence was capped at 100% for all analyses [20, 34]. Because this is a proof-of-concept study, we examined descriptive information and used basic statistical tests (e.g., paired t-tests) to examine pre-post changes in electronically-monitored and self/parent-reported adherence. We also assessed correlations between baseline JIA-specific knowledge, disease activity, and complexity of medication regimen in regard to adherence rates. Feasibility and acceptability of the intervention was analyzed via means and standard deviations and percentages of responses in the acceptable range.

## Results

### Study population and demographics

**Fig. 1:**
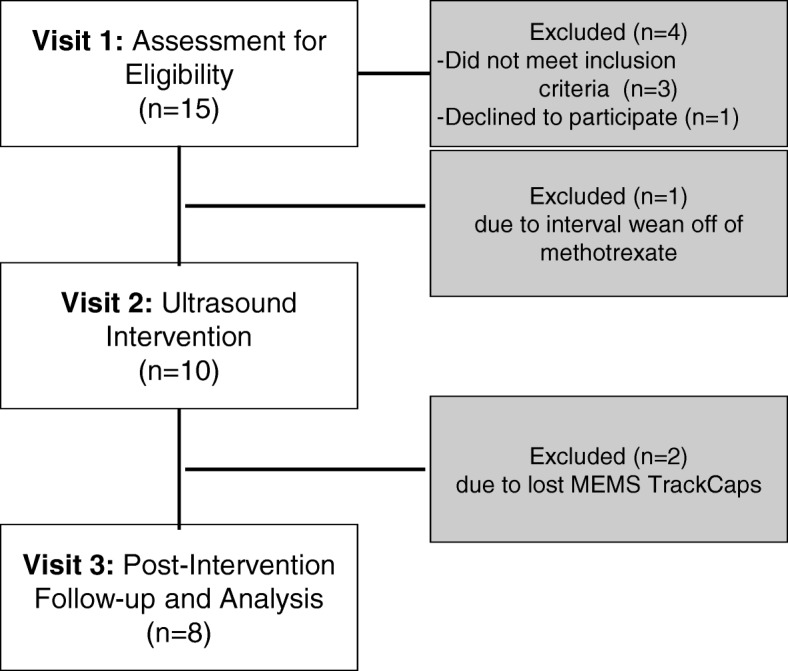
CONSORT Diagram

Figure 1 depicts our consort diagram of enrollment. Our patient sample included both polyarticular (80%) and extended oligoarticular (20%) subtypes. As noted in Table 1, the average patient age in years was 12.70 ± 2.06 with the majority being Caucasian (90%) females (80%). Parental marital status varied in our sample with 60% reporting being married. All patients were treated with methotrexate, with oral tablets being the most common formulation prescribed (55%). Most patients were on a concomitant biologic medication (63%), with 50% of those being infusion therapies.

**Table 1:** Descriptive Statistics of Participants

Characteristic	Total Sample (*n* = 11)	Completed Analysis (*n* = 8)
Patient Age (mean ± SD)	12.70 ± 2.06	12.75 ± 2.31
Gender (female %)	80	80
Race (white %)	90	87.5
Parental Marital Status (%)		
Single	20	25
Married	60	62.5
Divorced	10	12.5
Arthritis Type (%)		
Polyarticular RF-negative	80	75
Extended oligoarticular	20	25
Disease Duration (years)	3.9 ± 3.37	3.0 ± 3.05
Methotrexate route of administration (%)		
Oral	55	62.5
Subcutaneous	45	37.5
Concomitant Biologic (%)		
None	37	25
Etanercept	9	12.5
Adalimumab	9	12.5
Infliximab	27	37.5
Tocilizumab	18	12.5
Oral Steroids (%)	20	25

### Feasibility and acceptability

Feasibility of the study was based on full participation in the intervention and retention in the study until the final post-intervention visit. As seen in Fig. 1, of the 15 participants recruited, only one declined to participate. All of the eligible patients completed the intervention. Eight patients completed the entire study and were successful in returning the MEMS TrackCaps; however, two patients did not return their MEMS TrackCaps.

As noted in Table 2, based on a five point Likert Scale, the intervention was rated as comfortable (child M = 4.88 out of 5; parent M = 4.63) and a majority of patients felt that the ultrasound was helpful (child M = 4.25; parent M = 4.25), could benefit other children with arthritis (child M = 4.38; parent M = 4.75), and was explained in a clear way (child M = 4.63; parent M = 4.63). Lower acceptability ratings were noted in the ability of the intervention to change the way the family took their medications (child M = 2.63; parent M = 3.625) and change in their overall confidence in their care (child M = 3.5; parent M = 4.0). Most patients and parents were glad they participated in this study (child M = 4.5; parent M = 4.5).

**Table 2:** Descriptive Data of Feasibility and Acceptability Questionnaire (*N* = 8 Children and 8 Parents)

Title Items	Child N in the Ideal Range^a^	Parent N in the Ideal Range^a^
The ultrasound was not painful or uncomfortable.	7	8
The ultrasound provided me with new information.	5	7
The ultrasound provider explained the exam in a clear way.	6	8
The information that I learned from the ultrasound was helpful to me.	5	7
The ultrasound findings change the way we take medications.	2	4
I believe other children could benefit from ultrasounds in clinic.	5	8
The ultrasound made us feel more confident in our care.	4	4
I value the feedback I received from the MEMS TrackCaps.	6	6
Overall I am glad we participated.	7	7

^a^Based on a 4–5 score on a 5 point Likert Scale denoting agree or strongly agree. Assumption is made that a rating in this range notes a high rate of acceptability by respondents

### Intervention preliminary efficacy

**Fig. 2:**
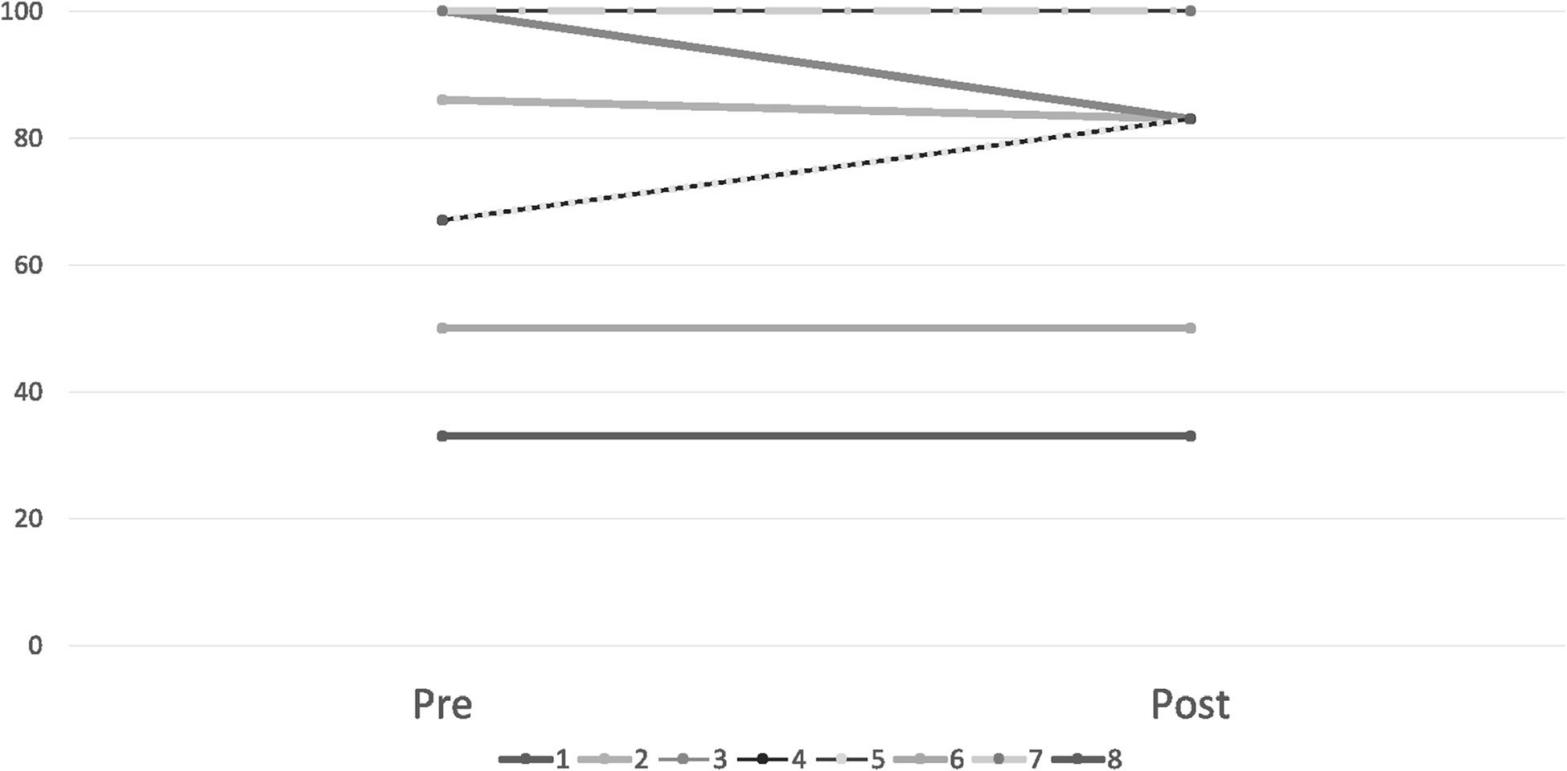
Participant Adherence Trajectory. Legend: Adherence is represented as the percentage of methotrexate dosages administered via MEMs TrackCap over the total prescribed treatment regimen doses. Adherence goal range is classically above 80%. Dashed lines represent overlapping participant data

Mean adherence at baseline was 75.3%, of which three patients had 100% adherence. Using a traditional 80% goal adherence level [33], 50% (*n* = 4 of 8) of patients were classified as “non-adherent” to methotrexate based on electronic monitoring at baseline. Each participant’s adherence data from pre to post-intervention is shown in Fig. 2. Two participants improved, four maintained adherence over time, and two participants declined. No significant differences were found from pre to post intervention for the sample for electronically-monitored adherence (t (df) = − 0.45, *p* = 0.67), self-reported adherence (t (df) = 1.35, *p* = 0.90) or parent-reported adherence (t (df) = 1.75, *p* = 0.12); See Table 2.

The number of adherence barriers decreased slightly for adolescents but increased slightly for parents (see Table 3). Specific examination of individual barriers (i.e. forgetting, pain, embarrassment etc.) revealed no major changes based on type.

**Table 3:** Pre- and Post- Comparative Statistics

Measure	Pre	Post	Mean % Change	Significance
% Adherence	75.34	77.06	1.73 ± 10.78	0.665
JIA Knowledge Score %	74.1	86.5	12.38 ± 15.17	0.054
Self-Report Adherence (Child)^a^	86.5	87.2	0.07 ± 1.31	0.890
Self-Report Adherence (Parent)^a^	88.0	80.8	−0.76 ± 1.74	0.124
Adherence Barriers Count Child^b^	6.75	3.13	−3.63 ± 5.88	0.125
Adherence Barriers Count Parent ^b^	4.88	5.38	0.50 ± 4.60	0.767
cJADAS^c^	12.13	6.13	−6.0 ± 10.00	0.133
Active Joint Count	7.38	2.50	−4.87 ± 6.10	0.058
Provider Global Assessment^d^	1.83	0.75	−1.06 ± 1.61	0.105
Patient Global Assessment^d^	3.19	2.88	−3.13 ± 2.25	0.706
PedsQL Parent Total Score (rg 0–100)^e^	76.98	76.28	−0.70 ± 17.72	0.934
Average Pain (VAS rg 0–10)	4.25	3.25	−1.00 ± 2.20	0.240

^a^PARQ/CARQ- Visual Analog Scale (VAS)– maximum 100 mm

^b^BAT- Represented as N out of a maximum 54 barriers

^c^cJADAS – Range 0–40, based on provider and patient global assessment and a 10 point joint exam

^d^Provider/Patient Global Assessment – VAS (0–10) with 0 being low disease activity

^e^PEdsQL scoring based on 5 point functional assessments. Range 0–100, with 100 being optimum functioning

### Intervention findings and clinical impact

**Fig. 3:**
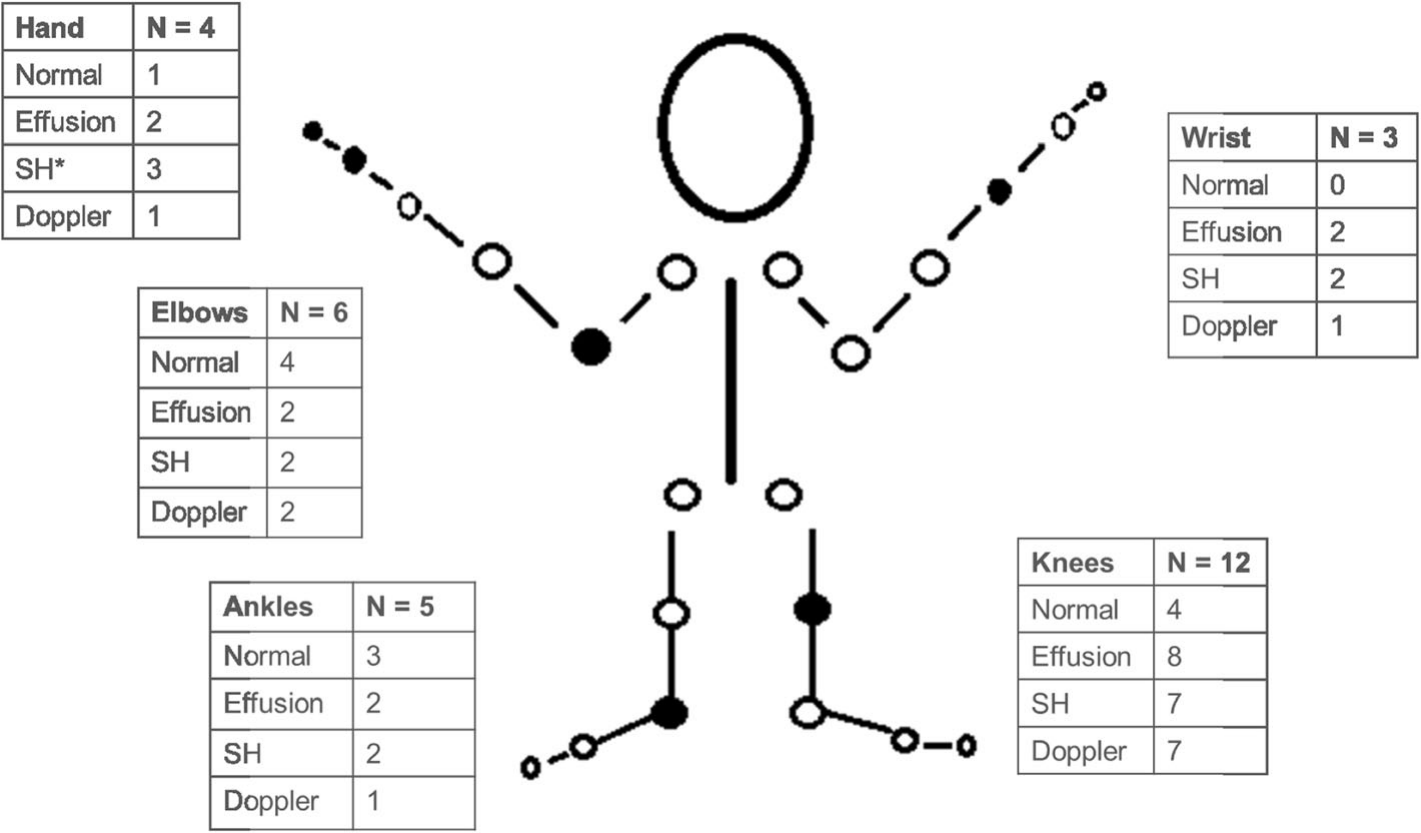
Ultrasound Intervention Findings. Legend: This figure demonstrates the location and description of the ultrasound findings by joint. SH- Synovial Hypertrophy

At least three joints were examined per participant, with 27 joints examined overall for the sample. Eighteen (66.7%) of the examined joints displayed abnormalities on ultrasound examination (see Fig. 3 for specific findings). Of note, there was a 63% discrepancy between the documented rheumatologist’s physical examination and the ultrasound exam, with the ultrasound picking up more joint pathology compared to physical examination. In total, four (50%) patients received a therapeutic change based on the ultrasound findings, including three new biologic starts.

No statistically significant differences were found on disease activity indices (i.e., active join count and provider and patient global assessment); however, trends in improvement were demonstrated (See Table 3). Further, no changes in HRQOL were found.

Post-hoc analyses indicated that the three patients that received a biologic treatment change had improvements in their disease activity scores, with average decreases ranging from high to moderate disease activity, which is clinically meaningful. Further patient-level comparative data is noted in Table 4.

**Table 4:** Patient-level Comparative Statistics

	Intervention			Characteristics						Outcomes			
	Abnormal U/S	Discrepant exams	New Biologic Started	MEMs Adherence (%)		Pt Self-reported Adherence^a^		Pt Number of Adherence Barriers		Joint Count		cJADAS	
				Pre	Post	Pre	Post	Pre	Post	Pre	Post	Pre	Post
1	Yes	Yes	No	33.3	33.3	52	79	10	4	0	0	3	3
2	Yes	Yes	Yes	86	83.3	92	97	4	6	6	0	8	6.5
3	Yes	Yes	Yes	100	83.3	93	94	10	7	7	3	28	11
4	Yes	Yes	Yes	66.7	83.3	93	87	18	2	8	3	15	7
5	No	No	No	100	100	100	100	0	1	0	0	3	3
6	Yes	Yes	No	50	50	80	77	2	3	12	5	3.5	6
7	Yes	No	No	100	100	87	88	2	0	0	1	0.5	1.5
8	Yes	No	No	66.7	83.3	95	75	8	2	26	8	20	11

Individual level data is demonstrated for intervention results, adherence pre and post comparisons and joint activity outcomes

^a^Based on CARQ responses on a 100 mm VAS

## Discussion

This proof-of-concept study was designed to test a MSKUS education-based adherence intervention amongst adolescents with JIA and their caregivers. Innovative aspects of the current study includes the novel multi-modal method used to demonstrate oral and injectable methotrexate adherence rates in this population, and the application of a rheumatology provider-based educational intervention for adolescents and their caregivers within the clinic setting.

Based on our retention rate of 80% and intervention completion rate of 100%, our study design and intervention methods appear to be feasible. The majority of participants found the ultrasound intervention to be clear, helpful and beneficial. Additionally, the MEMS TrackCap was an acceptable form of adherence measurement with no malfunctioning or missing data noted in the caps that were returned. Further, it was able to capture medication-taking behavior of both formulations of methotrexate, which is a significant strength of the study. To our knowledge, this is the first time MEMS TrackCaps have been used to measure adherence to injectable arthritis medications in pediatric rheumatology. Although adherence rates were capped at 100% for analyses, it is important to note that one family was actually found to have administered more than the prescribed regimen based on the patient living in two households due to divorce. That particular family valued the use of the TrackCaps for added accountability.

While mean adherence did not change significantly from pre to post intervention and correlations cannot be made based on the study size and scope, maintenance and improvements in patient self-reported and electronically-monitored adherence were observed in some patients. Additionally, the percentage of patients with “good” adherence (> 80%) [35] rose from 50 to 75% percent. Given that adherence typically declines over time [36], improvements and/or maintenance when adherence is already high is considered successful. Notably, 50% of our patients were non-adherent at baseline, which is consistent with the larger literature [23, 34], indicating that adherence is a significant issue for these patients.

Our data assessing self-reported and parent-reported adherence is consistent with prior studies suggesting inflation or over reporting. This may reflect response bias or pressure around social desirability. Prior data assessing self-reported adherence for adolescents with JIA (*N* = 116) on methotrexate found a median adherence of 98% [37]. Additionally, parent perceived adherence using the PARQ over a one year time period reported similarly high rates, varying between 86.1 and 92.0% [38]. In that study, the most highly correlated factor predicting higher self-reported adherence was believing that the therapy was helpful. This highlights the fact that patient beliefs influence the way self-management behaviors are performed and may be further evidence of the importance of standard patient education, with ultrasound being one method [39].

Perhaps the most salient aspect of this study design is the delivery of the intervention by a rheumatologist compared to other healthcare providers. The majority of JIA-focused adherence interventions were prior to biologic therapies and/or utilized psychologists, nurses or peer-mentors to perform the interventions [6, 40–42]. Since the advent of biologics, families of children with JIA have navigated more complex treatment regimens. Our prior work studying adherence barriers in JIA indicated that worry about JIA treatments was common for caregivers and patients [10], which suggests a need for more communication regarding specific concerns. Further, in a study assessing perceptions of adolescents prescribed biologic therapy, most adolescents preferred an active and involved role with dedicated education during their clinic visits [43]. Real-time, objective ultrasound interventions performed by rheumatologists, informs the patients of up-to-date disease status and presents the opportunity for additional questions or concerns regarding care. In some cases, it also justifies the need for therapy in the absence of perceived symptoms as well as the reverse. Our intervention not only included a review of normal joint anatomy and how ultrasounds can be useful in JIA, but patients were encouraged to actively participate in the ultrasound examination by seeing exactly what fluid or synovitis looks like in their own joints. This prompted many patients to have further discussions with their rheumatologists following the examination with a new sense of knowledge that they would have lacked prior. Previous work has established that both patients and providers value the information provided by ultrasounds [44]; however, more research is needed to measure the potentially compound effects that the receipt of an ultrasound can provide.

One unanticipated finding from this study was the benefit of adjunctive MSKUS for clinical care of patients with JIA. Despite the study being short-term, MSKUS aided in the detection of subclinical disease and informed therapeutic change. In our sample, the rate of abnormalities on ultrasound examination was 87.5% with two-thirds of those being discrepant to the rheumatologist’s physical examinations. Our rate of subclinical disease discovery was higher than prior study in pediatrics [15]. There was no recruitment screening done for patients presumed to be in active disease; thus, many of the rheumatology providers were surprised by the ultrasound findings. Outcomes in our sample revealed improvement overall, likely related to attainment of disease control based on optimized medication therapy. This is further supported by the fact that in the subset of patients that were started on a biologic during the intervention visit, the interval improvement in cJADAS score was more clinically meaningful as compared to patients that did not incur a therapeutic change. While unable to be correlated during this study, uncovering subclinical disease is one clear factor that could help to engage patients to adhere more closely to therapies and activate providers to screen for nonadherence more vigorously. This was mentioned by several rheumatologists upon hearing the results of their patient’s ultrasounds in our study.

As this was a proof-of-concept study, there are several important opportunities for improvement. Our small sample size was composed of a heterogeneous collection of participants including differences in therapy regimens, duration of disease and disease severity. Standardizing these parameters, increasing the sample, and testing the intervention via a randomized-controlled trial would increase our ability to detect the impact of our intervention on outcomes. An attention control arm including a verbal-education only group would aid in assessing the true effect of ultrasound imaging as an educational intervention. Also, the inclusion of two participants that received their intervention despite having a baseline adherence rate of 100% may have altered results due to ceiling effects. Any subsequent work in this area should exclude patients who demonstrate high adherence and thus do not need adherence interventions. Adherence interventions in other disease populations have successfully used this approach [32, 45]. Additionally, our short study duration of 12 weeks may have not provided sufficient time to examine behavioral changes over time. A longer study duration would also better demonstrate changes in our secondary outcomes (i.e. HRQOL and cJADAS) as these typically require longer monitoring to display changes over time. Furthermore, the medication regimen changes made during the study in several patients, may have altered the treatment routines of patients, thus potentially adding or subtracting barriers to adherence. However, the ethical need to respond to new information provided by the ultrasound, precludes avoidance of these therapeutic changes. Additional specific improvements that could be made to the ultrasound intervention itself include multi-provider blinded testing as to decrease biases imparted by the study team.

This study emphasizes the continuing importance of the development of adherence interventions for use in JIA. Following more rigorous testing, a multimodal adherence intervention including visual demonstration of disease using ultrasound could become common practice as either primary or secondary adherence promotion. There is still much work to be done in order to understand and encourage our patients to better navigate self-care and participate in their health. As providers of children with chronic illnesses in the midst of advancing technologies, we must continue to seek solutions to better bridge the patient-provider gap and deliver education to improve outcomes.

## Conclusions

Our study highlights an additional beneficial and novel use of MSKUS as a patient education tool. In this proof-of-concept trial, patients and families saw the purpose and helpfulness of real-time disease demonstration via ultrasound. We document that quantitative, electronically-measured adherence to methotrexate is, not only less than self-reported measures, but also below acceptable thresholds for therapy. We have also contributed to growing literature that establishes the utility of MSKUS as a useful clinical tool, with the potential to be harnessed for use in patient activation.

## Abbreviations

BAT: Barriers assessment tool
CARQ: Child adherence report questionnaire
cJADAS: Clinical juvenile arthritis disease activity score
DMARD: Disease modifying anti-rheumatic drug
JIA: Juvenile idiopathic arthritis
MEMS: Medication event monitoring system
MSKUS: Musculoskeletal ultrasound
PARQ: Parent adherence report questionnaire
PD: Power Doppler
PedsQL: Pediatric quality of life inventory
RA: Rheumatoid arthritis
SH: Synovial hypertrophy
VAS: Visual analog scale
